# New approaches in the neuropsychological evaluation of aADHD

**DOI:** 10.1192/j.eurpsy.2024.787

**Published:** 2024-08-27

**Authors:** L. I. Birtalan, S. Bálint, T. Kilencz, J. M. Réthelyi

**Affiliations:** ^1^Department of Psychiatry and Psychotherapy, Semmelweis University, Budapest, Hungary

## Abstract

**Introduction:**

In Hungary, the understanding and diagnosis of adult attention-deficit/hyperactivity disorder (aADHD) are influenced by a blend of international epidemiological data and the standardized criteria established in DSM-5. The diagnostic protocols at our aADHD Outpatient Clinic at Semmelweis University have been carefully adjusted and validated to align with the practical application of empirical evidence and the extensive clinical expertise of professionals. The current diagnostic protocol encompasses the use of diagnostic interviews (symptoms identification based on DSM criteria; SCID-5-PD; M.I.N.I.-PLUS-5.0), the Conners’ Adult ADHD Rating Scales–Self Report questionnaire (CAARS), heteroanamnesis with parents, a comprehensive neuropsychological instruments battery (including the Rey Auditory Verbal Learning Test, Stroop Test, Conners-CPT3, Trail Making Test) and WAIS-IV Intelligence Scale.

**Objectives:**

A valid and appropriate diagnosis plays a crucial social role by legitimizing individuals’ attention/health issues, confirming their concerns, and addressing cultural and moral expectations. The primary objective of this work is to refine the diagnostic methodology by extensive review of the international literature and the analysis of our own data.

**Methods:**

With the aim of aggregating and analyzing the collected data based on examinations of the Hungarian adult population, our assessment methods are employed to acquire detailed information regarding ADHD prevalence, symptoms, and the related neuropsychological profiles.

**Results:**

While various diagnostic approaches generally demonstrated good alignment, in some cases, significant discrepancies between neuropsychological assessment and the rest of our tools were observed, indicating a number of instances of false positives or false negatives. Especially the relevance of Rey Auditory Verbal Learning Test and Trail Making Test are questionable.

**Conclusions:**

The results highlight the necessity for more refined diagnostic criteria and a meticulous selection of neuropsychological techniques to enhance consistency between various approaches, ultimately enabling a more robust diagnostic accuracy.

**Disclosure of Interest:**

None Declared

